# Effects of Inhalation of Ether in Parturition

**Published:** 1847-06

**Authors:** 


5. *Effects of Inhalation of Ether in Parturition.*—The following extract of a letter to the editor of the *Provincial Med. and Surg. Jour.*, dated Paris, Feb. 25th, will be read with interest:

"The mind of the profession here is entirely occupied upon the ether question, to the temporary exclusion of all others. The Baron Dubois read a very interesting paper to the Academy of Medicine the day before yesterday, giving the details of six cases of protracted and difficult labors, in which the vapor of sulphuric ether was inhaled with marked advantage. The particulars will be almost immediately published, but in the interim I may as well tell you that the results of the baron's experience warrant him in concluding that the vapor of the ether may be inhaled by parturient women:—1st, without any danger to mother or child; 2d, with advantage to both, in so much as that it destroys all resistance in the voluntary muscles of the perineum, relaxing or rather paralyzing them for the moment, without impeding or interfering in the slightest degree with the natural physiological muscular actions of the uterus. The baron has also observed that the abdominal muscles in their actions in parturition are not at all affected by the inhaled ether.

"The two first cases—both instrumental—one in labor forty hours, the other thirty-six before the vapor was inhaled—turned out ultimately unfortunate, as both patients died of puerperal fever which was at the time prevailing in the hospital (La Maternite). This sad result, the baron does not think can be ascribed at all to the use of the vapor; nor does he on the other hand attribute the immunity of the other patients in the same hospital to it.

"The baron, upon interrogating the patients after delivery, as to their sensations during the operation, was informed by all but one, that they felt nothing of what was doing, but that one smiled and would not say what she had felt. It afterwards turned out that this patient, by her confessions to the nurse, was ashamed to say what she felt and thought, as she found herself engaged, all the time when under the influence of the ether and undergoing the operation of delivery, with her husband in that preliminary process which is so essential to the bringing about of that condition in which ladies like to be who love their lords."—*Med. News.*

